# DCAF2 is essential for the development of uterine epithelia and mouse fertility

**DOI:** 10.3389/fcell.2024.1474660

**Published:** 2024-09-19

**Authors:** Man Yang, Kaixuan Wang, Liang Zhang, Hongya Zhang, Cong Zhang

**Affiliations:** ^1^ Center for Reproductive Medicine, Ren Ji Hospital, School of Medicine, Shanghai Jiao Tong University, Shanghai, China; ^2^ Shandong Provincial Key Laboratory of Animal Resistance Biology, College of Life Sciences, Shandong Normal University, Jinan, Shandong, China; ^3^ Research Center of Translational Medicine, Jinan Central Hospital Affiliated to Shandong First Medical University, Jinan, Shandong, China; ^4^ Shanghai Key Laboratory for Assisted Reproduction and Reproductive Genetics, Shanghai, China

**Keywords:** DCAF2, epithelium, estrogen receptor, infertility, progesterone receptor

## Abstract

**Introduction:**

The successful outcome of a pregnancy depends on the proper functioning uterine epithelium. DNA damage binding protein 1 and cullin 4-associated factor 2 (DCAF2), a conserved substrate receptor for the cullin 4-RING E3 ubiquitin ligase (CRL4) complex, is essential for maintaining genome stability by facilitating ubiquitin-mediated degradation of substrates.

**Methods:**

To better understand the physiological role of DCAF2 in female reproduction, we conducted a study using mice with conditional knockout (cKO) of DCAF2 in the uterus using the progesterone receptor Cre (*Pgr* ^Cre/+^) mouse model.

**Results:**

Our results showed the cKO mice were completely infertile, despite having ovarian function. The cKO mice exhibited severely thin uteri, demonstrating notable defects in both the uterine epithelium and a lack of glands. In addition, there were impaired proliferation and differentiation of epithelial cells in the cKO mice, ultimately resulting in failed implantation. Moreover, through deciphering the uterine transcriptome of cKO mice, we revealed crucial differentially expressed genes associated with steroid signaling. Further experiments have demonstrated cKO mice exhibit elevated uterine PGR signaling and reduced estrogen receptor signaling, although the levels of progesterone and estrogen remained unaltered. These alterations may contribute to defects in epithelium.

**Discussion:**

Overall, our findings highlight a previously unrecognized but indispensable role for DCAF2 in the development of uterine luminal and glandular epithelium by orchestrating PGR and estrogen receptor responses. Its deficiency in the uterus leads to mouse infertility.

## Introduction

The uterine endometrium, where the embryo resides and grows, contains a single layer of luminal epithelium (LE) surrounded by stromal cells, as well as multiple coiled glands lined by simple glandular epithelium (GE) (Cui et al., 2017). The LE is the initial site of contact between the mother and the invading blastocyst. In mouse, blastocysts home and attach to the LE within specialized implantation crypts that derive from epithelial evaginations (Kim et al., 2023). Clearly, the exact organization of the LE is crucial for establishing a successful pregnancy. Disruption of epithelial development often leads to infertility in the adult mice (Spencer, 2014; Jeong et al., 2010).

The glands can be seen as the derivatives of LE. Optimal proliferation of the LE is crucial for the development of gland. This process, known as adenogenesis (Kobayashi and Behringer, 2003), typically begins postnatally in mice. Within 3 days of birth, the uterine epithelium showed an oval lumen along the mesometrial-antimesometrial axis. By 5 days old, the uterine lumen has increased complexity in shape, accompanied by epithelial evaginations into the surrounding mesenchyme, a stage called bud formation, which marks the initiation of gland development. At 10 days old, the uteri show the extension of glands from the LE into the surrounding stroma. By 15 days, the basic structure of the uterus is established, with further increased folding of the LE and an increased number of glands. At 8 weeks old, the adult uteri continue to grow in size and complexity while maintaining the same basic structure. The number of uterine glands further increases, resulting in the establishment of an intricate and highly organized gland network throughout the stroma.

The spatio-temporal organization of the epithelium, including adenogenesis, is implicated in series of cell proliferation and differentiation events. These events are controlled not only by intrinsic regulatory mechanisms within the uterus but also by extrinsic factors, particularly the steroid hormones estrogen (E2) and progesterone (P4) from the ovary (Cui et al., 2017). E2 drives the proliferation of uterine epithelia by binding to the estrogen receptor (ER), while P4 blocks E2-enhanced proliferation of epithelia through the progesterone receptor (PGR) (Li et al., 2011; Zhang S. et al., 2013). E2 and P4 have always been the top priority in insights into reproductive disorders (Wang and Dey, 2006). However, the interplay between intrinsic regulators and steroid hormones during the development of uterus remains enigmatic and needs to be explored.

UV-damaged DNA-binding protein 1 and cullin 4 associated factor 2 (DCAF2), also known as CDT2 or DTL, is a substrate receptor for the cullin 4-RING E3 ubiquitin ligase (CRL4) complex (CRL4^DCAF2^) (Higa et al., 2006). The CRL4^DCAF2^ complex, with DCAF2 as its main component, recognizes and degrades substrates involved in DNA replication and the cell cycle, including CDT1, P21, CHK1 and PR-Set7. This complex plays crucial roles in maintaining genome stability (Abbas and Dutta, 2011). On the whole, DCAF2 is essential for the survival of cells. Earlier study has showed *Dcaf2* knockout mice die at the preimplantation stage (Liu et al., 2007). Later, the application of conditional knockout (cKO) strategy has provided insight into the *in vivo* function of a gene. Depleting DCAF2 in oocytes arrests the development of embryos during the first cell cycle in mice (Xu et al., 2017). Our previous study revealed the ablation of DCAF2 in trophoblast cells leads to failed early placentation, and consequent embryonic death at gastrulation owing to excessive DNA damage and resulting cell apoptosis (Yang et al., 2022). Nevertheless, it has remained unclear whether and how uterine DCAF2-mediated signaling regulates the development and function of the uterus. In this study, we aimed to elucidate the role of DCAF2 in the uterus by deleting *Dcaf2* in the uterus using a mating strategy involving progesterone receptor Cre (*Pgr* ^Cre/+^) mice and mice carrying *Dcaf2* alleles flanked by *loxP* sequences (*Dcaf2* ^fl/fl^). We found the deletion of *Dcaf2* led to developmental defects in the LE and a lack of glands in the uterus, which affected the function of uterus and resulted in female infertility. In addition, the levels of E2 and P4 remained unchanged in the cKO mice at 8 weeks old, but there was an increase in uterine PGR signaling increased and a reduction in ER signaling, which contributed to the developmental defects in the epithelium. These findings suggest that DCAF2 is crucial for uterine development and function via orchestrating PGR and ER signaling.

## Materials and methods

### Mice and treatments

Mice used in this study were maintained on a C57BL/6J genetic background. The mice were housed at the Experimental Animal Center of Shandong Normal University under a 12 h light/dark cycle. All animal experiments were approved by the Animal Ethics Committee of Shandong Normal University. Progesterone receptor Cre (*Pgr* ^Cre/+^) model and mice with floxed alleles of *Dcaf2* (*Dcaf2* ^fl/fl^) were produced as previously described (Xu et al., 2017). *Pgr* ^Cre/+^ mice were crossed with *Dcaf2* ^fl/fl^ mice to generate mice with conditional depletion of *Dcaf2* in the uterus (*Pgr* ^Cre/+^; *Dcaf2* ^fl/fl^, cKO). *Dcaf2* ^fl/fl^ mice from the same litter were used as control (Con). Genomic DNA isolated from tail tips was used for genotyping by PCR. For fertility analysis, Con (n = 4) and cKO (n = 6) female mice at 6 weeks of age were housed with fertile wild-type males for over 6 months. Mating was evidenced by the presence of vaginal plugs. The numbers of litters and pups during this period were counted and recorded.

### Timed gestation and visualization of implantation sites

For timed gestation, 8-week-old Con or cKO mice were crossed with fertile wild-type males. Vaginal plugs were checked at 07:00 daily, and the day when the plug was observed was recorded as 0.5 days post-coitum (dpc). Uteri were dissected at dpc 3.5 and 4.75, and fixed with 4% paraformaldehyde for histological analysis. Implantation sites at 4.75 dpc were visualized by injecting 1% Direct Blue Dye (Macklin, Shanghai, China) into the tail vein, and 5 min later the mice were sacrificed. The number of implantation sites, indicated by blue areas, was counted.

### Superovulation and hormone analysis

Immature female mice were intraperitoneally injected with 5 IU pregnant mare serum gonadotropin (Ningbo Sansheng Pharmaceutical Co, Ltd., Ningbo, China) at 17:00, followed by 5 IU human chorionic gonadotropin (Ningbo Sansheng Pharmaceutical Co, Ltd.) 46 h later (Zhang C. et al., 2013). After 18 h, the oviducts were dissected, and needle tips were used to tear the oviducts to release cumulus-oocyte complexes into M2 medium (Sigma-Aldrich, Saint Louis, MO, USA) containing hyaluronidase (Sigma-Aldrich) to separate out MⅡ oocytes. Then the numbers of MⅡ oocytes were counted under an anatomical microscope (Olympus, Southborough, MA, USA). For hormone analysis, blood was collected through cardiopuncture from 3.5 dpc mice. The serum was separated by centrifugation. The levels of P4 and E2 in the serum were analyzed as previously reported (Chang et al., 2017; Frantsiyants et al., 2020) using enzyme-linked immunosorbent assay (ELISA) kits (PROG ELISA Kit and E ELISA Kit, Cusabio, Wuhan, China) according to the manufacturer’s instructions. Both of the coefficients of variation (CVs) of intra- and inter-assay were less than 15%. The minimum detectable concentration of the kit for estrogen was 25 pg/mL, and for progesterone, it was 0.3 ng/mL.

### RNA isolation, qPCR, and RNA-seq analysis

Total RNA was isolated from whole uteri at 3.5 dpc using the TRNzol reagent (Tiangen, Beijing, China) according to the manufacturer’s guidelines (Wang et al., 2020; Zhang et al., 2016). The RNA was then reverse transcribed into cDNA using FastQuant RT Kit with gDNase (Tiangen). QPCR was performed to quantitate the mRNA levels using the SYBR Green PreMix (Tiangen) on a LightCycler 96 system (Roche, Basel, Switzerland). The qPCR data was analyzed using the 2^−ΔΔCt^ method (Zhang et al., 2018). The primer sequences used in this study are provided in Supplementary Table S1.

For RNA sequencing, total RNA was extracted from the uterus using Trizol reagent (Invitrogen, Carlsbad, CA, USA). The RNA quality was evaluated using the Agilent 2200. Only RNA samples with an RNA integrity number>7.0 were used for cDNA library construction. The cDNA libraries were constructed using the TruSeq Stranded mRNA Library Prep Kit (Illumina, Inc.) according to the manufacturer’s instructions. The libraries were quality-controlled using the Agilent 2200 system and sequenced using the NovaSeq 6000 platform in a 150 bp paired-end run.

For RNA-seq analysis, clean reads were obtained from the original reads by removing adapter sequences and low-quality reads before mapping. Then, the clean reads are aligned with the mouse genome (mm10, NCBI) using the Hisat2 program. Gene counting is done using HTseq, while gene expression was determined using the RPKM method. DESeq2 algorithm was used to filter DEG. After significance analysis, the *p*-value and FDR analysis needed to meet the following criteria: 1) fold change >2 or <0.5; 2) *p* < 0.05, FDR <0.05. Gene Ontology (GO) analysis was performed to elucidate the biological significance of DEG. Fisher’s exact test was applied to determine significant GO categories (*p* < 0.05).

### Histology, immunohistochemistry, and immunofluorescence analyses

The tissues isolated from mice at 3.5 dpc were fixed with 4% paraformaldehyde for 24 h. After fixation, the tissues were dehydrated using graded ethanol, ranging from 35% to 100%. Subsequently, they were hyalinized with xylene and embedded in paraffin. The embedded tissues were sectioned into 5 μm slices, mounted on slides, and subjected to deparaffinization and rehydration processes. For hematoxylin and eosin (H&E) staining, the sections were incubated with hematoxylin (Solarbio, Beijing, China) for 1 min, followed by a 5 min water wash. Then the slides were incubated with eosin for 2 min, washed, dehydrated in graded ethanol, and hyalinized in xylene. Finally, the sections were mounted using neutral gum with coverslips.

For immunohistochemical staining, the antigen was retrieved by immersing the sections in citrate buffer (Solarbio) in a pressure cooker. Then the endogenous peroxidase activity was eliminated using a 3% hydrogen peroxide solution. After three washes in PBS, the sections were blocked with blocking buffer (Solarbio) for 1 h at room temperature. Subsequently, the sections were incubated overnight with primary antibodies listed in Supplementary Table S2. After three more washes in PBS, the sections were incubated with the secondary antibodies (HA1001; HA1006, Huabio, HangZhou, China) at 1:400 dilutions at room temperature. After 1 h incubation, diaminobenzidine (ZSGB-BIO, Beijing, China) was used to visualize the antigen protein. The sections were successively counterstained with hematoxylin, dehydrated in graded ethanol, hyalinized in xylene, mounted using neutral gum, and finally imaged using a 3D digital scanner (Pannoramic MIDI, 3DHISTECH, Budapest, Hungary). The quantification of positive immunostaining was performed using Image-Pro Plus version 6.0 (Media Cybernetics, Silver Spring, MD, USA).

For double-immunofluorescence staining, the previous steps were identical to immunohistochemical staining. Then, the sections were co-incubated with anti-SMA and -CK7 antibodies (listed in Supplementary Table S2) diluted in antibody dilution buffer (Solarbio). After three more washes in PBS, the sections were co-incubated with TRITC (red) and FITC (green)-conjugated secondary antibody (Huabio) for 1 h in a dark place. The nuclei were counterstained with 4′,6-diamidino-2-phenylindole (DAPI), and mounted using an anti-fade mounting medium (Solarbio). The primary antibodies used for immunohistochemical or double-immunofluorescence staining were listed in Supplementary Table S2.

### Statistical analysis

GraphPad Prism version 8.0 (GraphPad Software, San Diego, CA, USA) was utilized for the statistical analysis. The data are presented as mean ± SEM. The experiments were repeated at least three times. The Student’s t-test was used to compare between two groups. *p* < 0.05 indicates statistically significant.

## Results

### The conditional ablation of *Dcaf2* in the uterus results in female infertility

**FIGURE 1:**
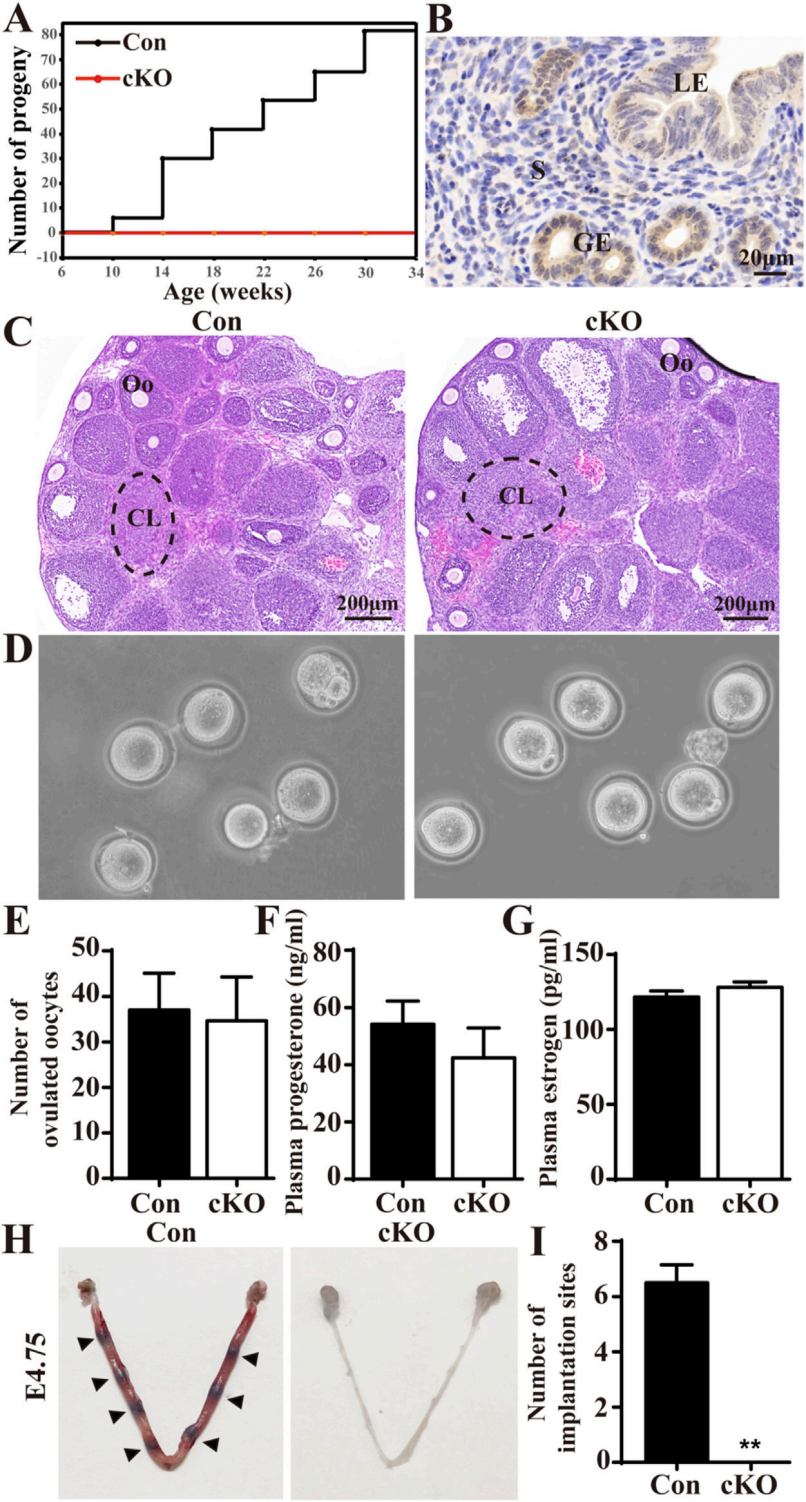
Conditional ablation of *Dcaf2* in the uterus results in female infertility. **(A)** The total number of pups produced by control (Con) and conditional knockout (cKO) females over a 6- month breeding period. **(B)** Immunostaining of DCAF2 in the uteri of Con mice. **(C)** Hematoxylin and eosin (H&E) staining of superovulated ovaries from Con and cKO mice, with dotted lines indicating corpus luteum. CL, corpus luteum; Oo, oocyte. **(D)** Microscopic analysis of MⅡ oocytes released from superovulated oviducts from Con and cKO mice (n = 4). **(E)** The number of MⅡ oocytes collected from superovulated Con and cKO mice (n = 4). **(F)** plasma progesterone (P4) levels at 3.5 days postcoitum (dpc) for Con and cKO (n = 4) females mated to wild-type males. **(G)** Plasma estrogen (E2) levels at 3.5 dpc. **(H)** Visualization of implantation sites at 4.75 dpc using direct blue dye, with arrowheads indicating implantation sites. **(I)** Quantification of the number of implantation sites in Con and cKO mice (n = 4). LE, luminal epithelium; GE, glandular epithelium; S, stroma. Scale bars: 20 and 200 μm. Data are expressed as mean ± SEM. **p < 0.01.

To evaluate the fertility of *Dcaf2* cKO females, we performed a fertility trial lasting over 6 months. This trial included four control (Con) females and six *Dcaf2* cKO female mice. All the mice were 6 weeks old at the start of the trial and were mated with fertile wild-type males. Throughout the trial period, all Con female mice exhibited normal mating behavior, as evidenced by the presence of vaginal plugs. They also successfully produced more than 80 pups (Figure 1A). In contrast, none of the *Dcaf2* cKO females showed any signs of pregnancy and no pups were observed, demonstrating complete sterility resulting from the uterine ablation of *Dcaf2*. Additionally, the immunohistochemistry study revealed DCAF2 was highly expressed in the uterus, particularly in the uterine epithelium (Figure 1B). These findings suggest that DCAF2 is required for female reproductive function.

Since PGR is present in ovarian preovulatory follicles in addition to the uterus (Soyal et al., 2005), we examined the ovarian function. Histological staining of superovulated ovaries revealed the presence of normal corpora lutea (CL) and oocytes (Oo) in both Con and cKO mice (Figure 1C). There were no significant differences in the morphology and number of oocytes between the two groups (Figures 1D, E). Additionally, we found no significant differences in the serum levels of P4 and E2 between Con and cKO mice at 8 weeks of age (Figures 1F, G). Therefore, the ovarian function was normal in the cKO mice. We also assessed the implantation of blastocysts. After injecting Direct Blue dye at 4.75 dpc, we observed normal implantation sites in Con mice, while no visible implantation sites were observed in cKO uteri (Figures 1H, I). Furthermore, the blastocysts flushed out from cKO uteri appeared normal (data not shown). Overall, our findings demonstrate that although the ovarian function and blastocysts were normal in cKO mice, these mice lacked implantation sites and were therefore infertile.

### *Dcaf2* cKO mice exhibit severe developmental defects in their uteri

**FIGURE 2:**
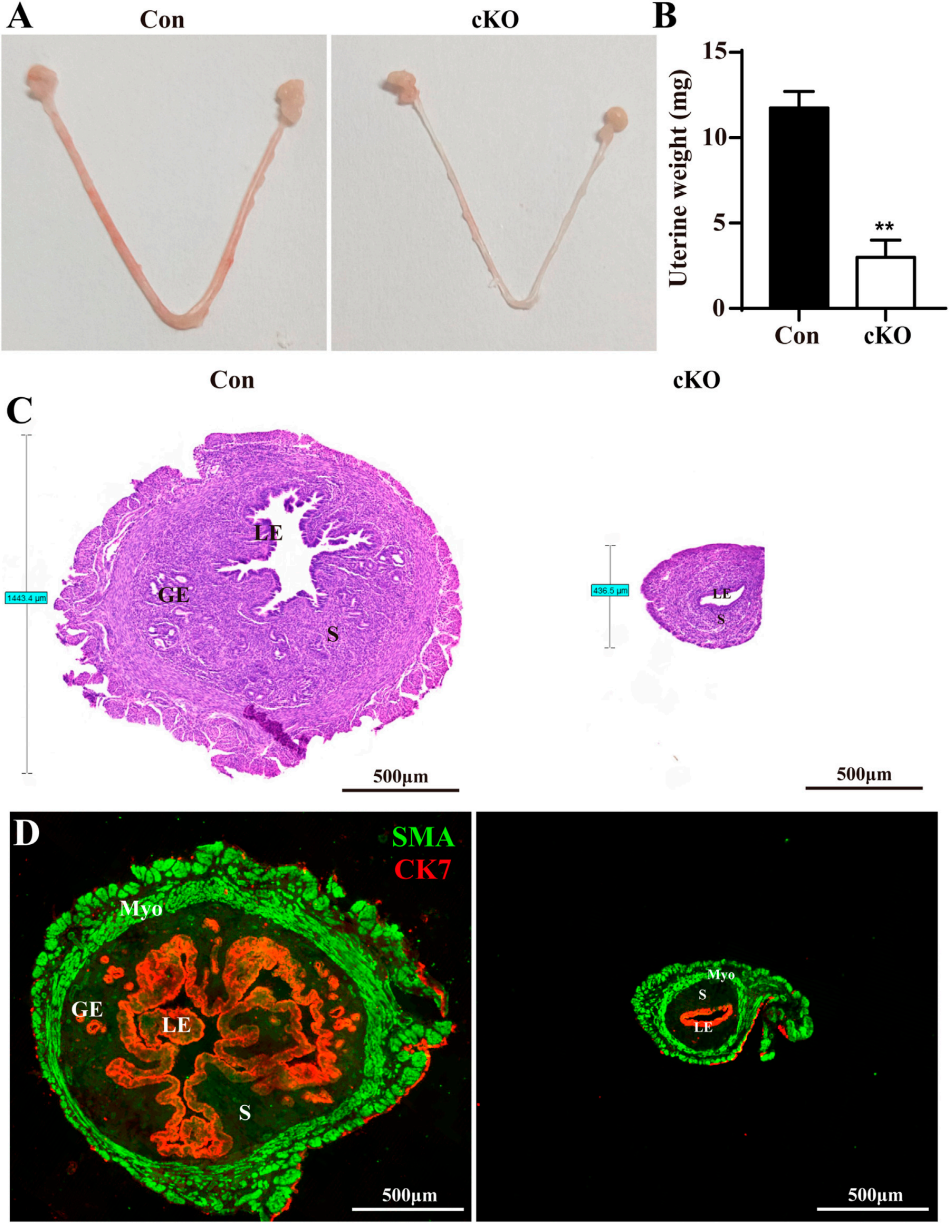
*Dcaf2* cKO mice show serious developmental defects in uteri. **(A)** Gross images of Con and cKO uteri at 8 weeks of age. **(B)** Uterine weight of Con and cKO uteri (n = 4). **(C)** H&E staining of the uteri from Con and cKO mice. **(D)** Double immunofluorescence staining with CK7 (red) and SMA (green) in the uteri from Con and cKO mice. LE, luminal epithelium; GE, glandular epithelium; S, stroma. Scale bars: 500 μm. Data are expressed as mean ± SEM. **p < 0.01.

To evaluate the development of Dcaf2-deficient uteri, we dissected uteri from postnatal day (PND) 5–42 in Con and cKO mice (Supplementary Figure S1). The cross section of the uteri showed that there was no difference in the structure of uterus from PND5 to PND15, except for the presence of glands in Con mice but not in cKO mice since PND15 and thereafter. Starting from PND28, cKO uteri were significantly thinner compared to Con uteri, and this difference increased with age. To better illustrate this phenotype, we selected 8-week-old mice for subsequent experiments. As shown in the gross uteri images in Figure 2A, cKO uteri displayed similar length but were considerably thinner than Con uteri when viewed under the same magnification. Additionally, cKO uteri weighed much less (Figure 2B). Histological analysis with H&E staining revealed that the thickness of cKO uteri was less than one-third of Con uteri (Figure 2C). Furthermore, double immunofluorescence using smooth muscle actin (SMA) and cytokeratin 7 (CK7) as markers for uterine myometrium and epithelium, respectively, was employed to characterize the uterine structure. The results indicated that the LE in cKO uteri was significantly thinner and displayed severe abnormalities, with the absence of glands (Figure 2D).

### *Dcaf2* depletion blocks epithelial proliferation

**FIGURE 3:**
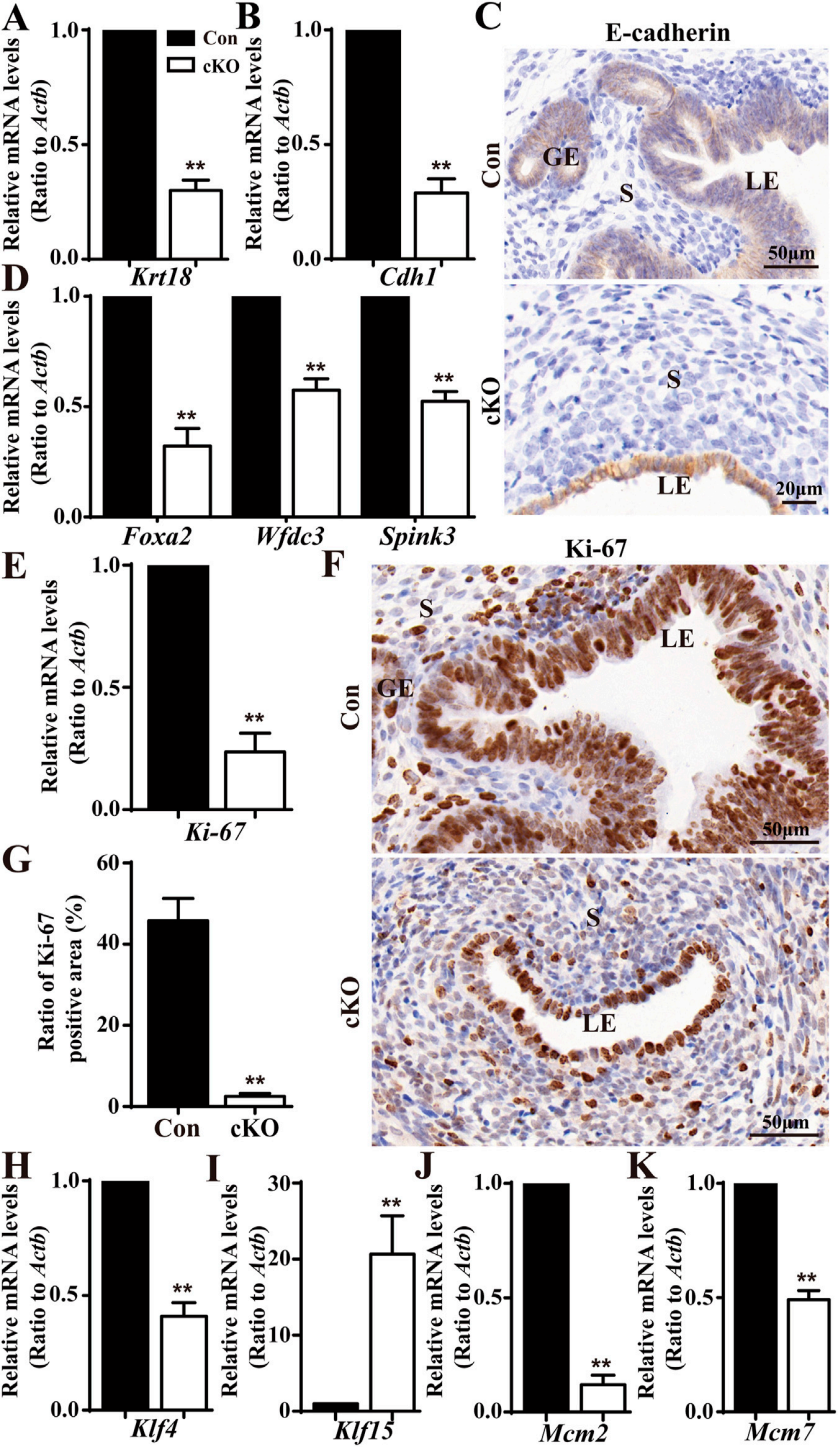
Deficiency in epithelial growth in the cKO uterus. **(A,B)** QPCR results showing a significant reduction in epithelial marker genes (*Krt18* and *Cdh1*) in cKO mice (n = 6), respectively. **(C)** Immunostaining with a marker of uterine epithelium, E-cadherin. **(D)** QPCR results showing significantly reduced gland-specific genes *Foxa2*, *Spink3,* and *Wfdc3* in the uteri of cKO mice (n = 6). **(E)** QPCR results showing a significant reduction in the proliferation marker gene *Ki-67* in cKO mice (n = 6). **(F)** Immunostaining of Ki-67 in the uteri of Con and cKO mice (n = 4). **(G)** Quantitation of Ki-67 positive cells in the uteri of Con and cKO mice (n = 4). **(H–K)** QPCR analysis of *Klf4* (encoding a transcription factor regulating the proliferation), Klf15, Mcm2(encoding a DNA replication licensing factor), and Mcm7 in the uteri of Con and cKO mice (n = 6), respectively. Scale bars: 50 and 20 μm. Data are expressed as mean ± SEM. **p < 0.01.

To illustrate the developmental defects in uterine epithelium, we conducted qPCR analysis of two epithelial markers, cytokeratin 18 (encoded by *Krt18*) and E-cadherin (encoded by *Cdh1*). We found both *Krt18* and *Cdh1* were significantly downregulated in cKO uteri compared to Con uteri (Figures 3A, B). Furthermore, immunohistochemistry anylysis displayed the expression of E-cadherin in the LE and GE of Con and cKO mice, clearly showing abnormal development of the LE in cKO mice (Figure 3C). Since glands was absent in cKO uteri, we also analyzed the levels of 3 GE marker genes: forkhead box a2 (*Foxa2*), serine peptidase inhibitor Kazal type 3 (*Spink3*), and WAP four-disulfide core domain 3 (*Wfdc3*). We observed that all three genes were reduced in cKO uteri (Figure 3D).

Given the observed epithelial defects observed in cKO uteri, we speculated there was insufficient proliferation in the epithelial cells upon *Dcaf2* depletion. To test this hypothesis, we performed qPCR analysis on the cell proliferation marker gene, *Ki-67*. As depicted in Figure 3E, *Ki-67* levels were significantly downregulated in cKO uteri. Furthermore, Ki-67 staining revealed a severe deficiency in epithelial proliferation in cKO uteri (Figures 3F, G). Previous studies have shown that cascades of transcription factors, such as Kruppel-like transcription factors KLF4 and KLF15, are activated to mediate and amplify the effects of E2 and P4 on the proliferation of uterine epithelium. These transcription factors coordinately regulate the genes involved in DNA replication events, including minichromosome maintenance (MCM) 2 and MCM 7, increased levels of KLF4 enhance MCM2 and MCM7 expression in response to E2, while increased levels of KLF15 inhibit MCM2 and MCM7 expression in response to E2 and P4 (Ray and Pollard, 2012; Knoedler and Denver, 2014). In our study, we observed reductions in *Klf4* levels and increases in *Klf15* levels (Figures 3H, I). Additionally, we found that levels of both *Mcm2* and *Mcm7* were reduced (Figures 3J, K).

### Elevated uterine response to P4 and decreased response to E2 upon *Dcaf2* depletion

**FIGURE 4:**
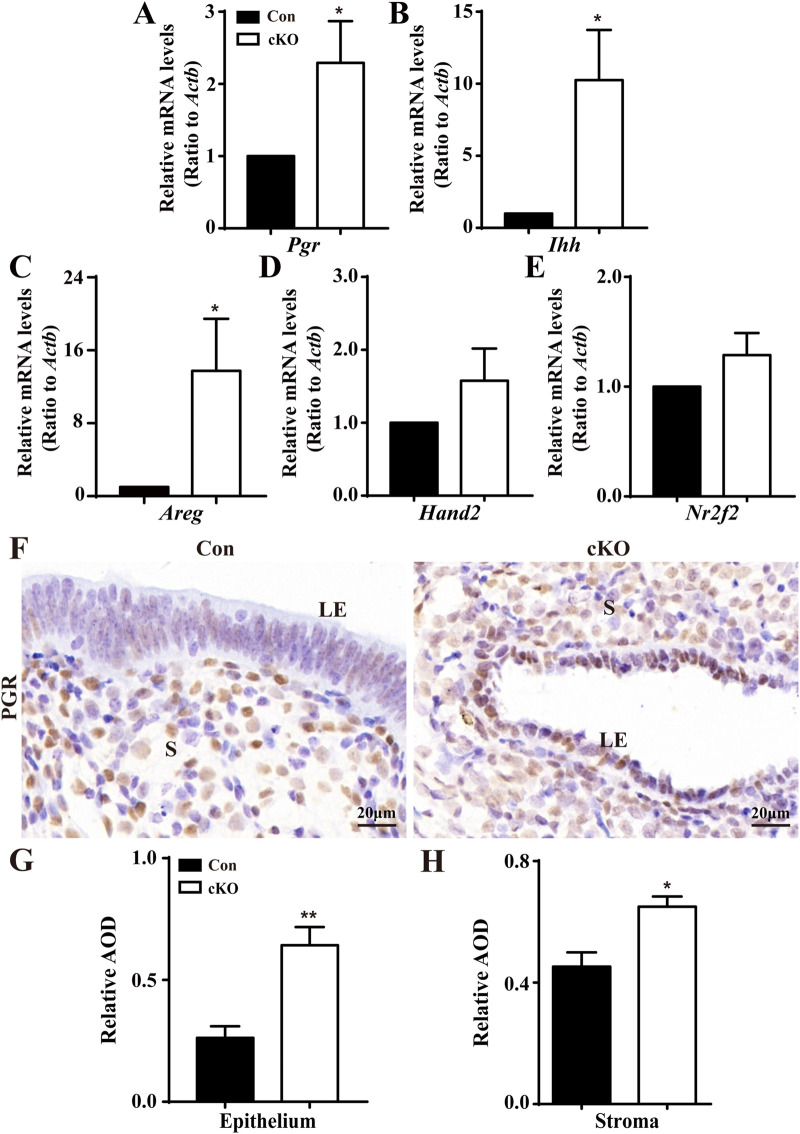
Abnormal upregulation of P4-regulated genes in cKO uteri during the window of implantation. **(A–E)** QPCR results showing the levels of *Pgr* and PGR-regulated genes *Ihh*, *Nr2f2*, *Hand2,* and *Areg* in Con and cKO mice (n = 6), respectively. **(F)** Immunostaining of PGR in the uteri of Con and cKO mice at 3.5 dpc (n = 4). **(G,H)** Quantification of the immunostaining of PGR (n = 4) in epithelium and stroma, respectively. AOD: Average optical density (IOD [Integrated Optical Density]/area). LE, luminal epithelium; S, stroma. Scale bars: 20 μm. Data are expressed as mean ± SEM. *p < 0.05; **p < 0.01.

To deciphering the underling molecular mechanism by which *Dcaf2* depletion results in the deficient proliferation in the uterine epithelium, we performed RNA-seq analysis of the global gene expression profiles of the uteri of Con and cKO mice (Supplementary Figure S2A). Further Gene Ontology analysis revealed differentially expressed genes (DEG) associated with steroid signaling between Con and cKO uteri (Supplementary Figure S2B). Considering the regulatory actions of P4 and E2 in the proliferation of uterine epithelium, we examined the levels of P4/E2-responsive genes. As shown in Figures 4A–E, the levels of *Pgr*, which encodes the progesterone receptor, and P4-responsive genes, Indian hedgehog (*Ihh*) and amphiregulin (*Areg*), were significantly increased in uteri after *Dcaf2* depletion. There was also an increase in the levels of other P_4_-responsive genes, such as chicken ovalbumin upstream promoter-transcription factor II (*Nr2f2*) and heart and neural crest derivatives expressed 2 (*Hand2*), although it did not reach statistical significance. Additionally, immunostaining revealed a significant increase in PGR expression in the uterine epithelium and stroma (Figures 4F–H).

**FIGURE 5:**
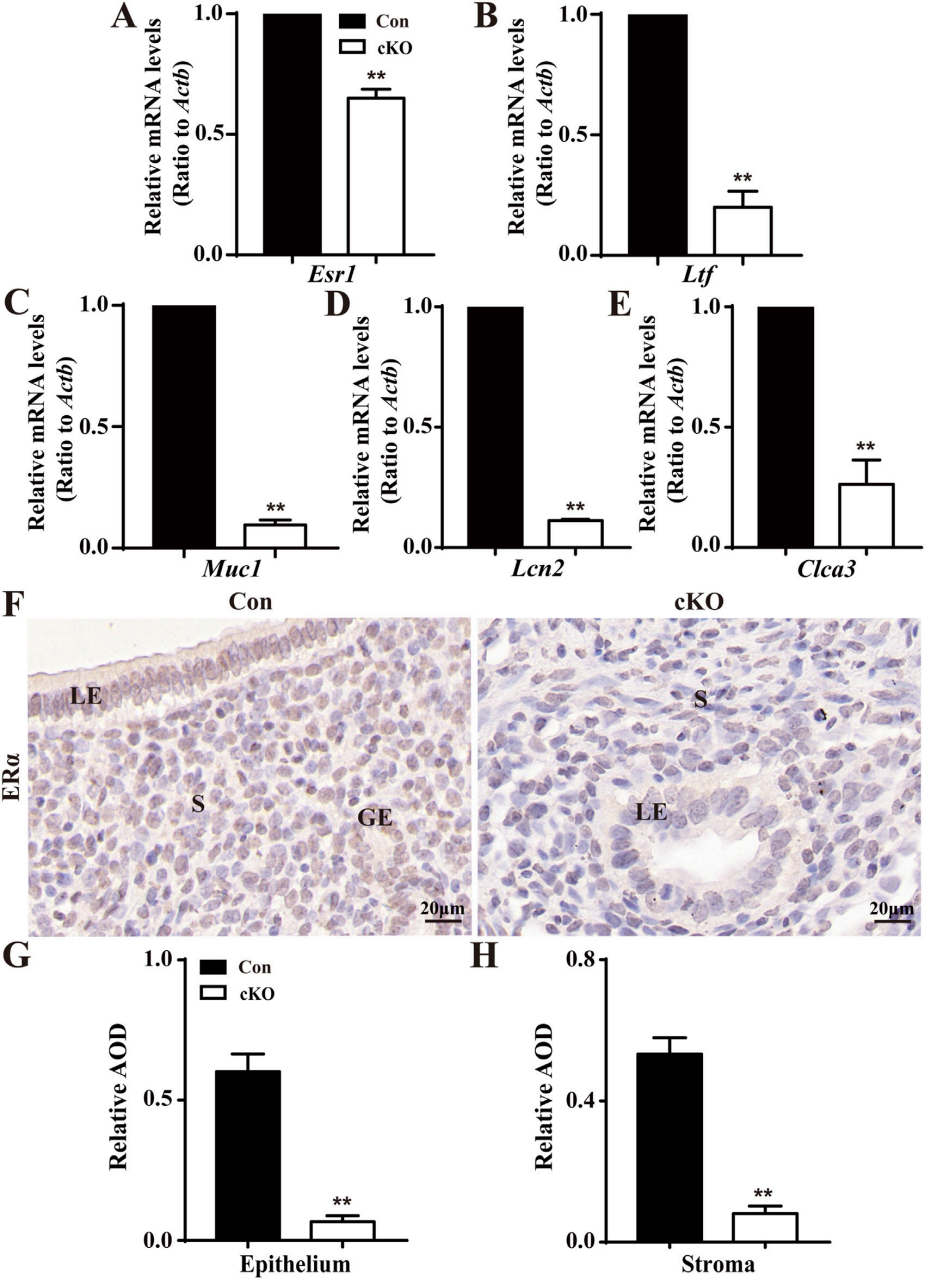
Reduced levels of E2-regulated genes in cKO uteri during the window of implantation. **(A–E)** QPCR results showing the levels of *Esr1* and ER-regulated genes *Ltf*, *Muc1*, *Lcn2,* and *Clca3* in the uteri of Con and cKO mice (n = 6), respectively. **(F)** Immunostaining of ERα in the uteri of Con and cKO mice at 3.5 dpc (n = 4). **(G,H)** Quantification of the immunostaining of ERα (n = 4), respectively. AOD: Average optical density (IOD [Integrated Optical Density]/area). LE, luminal epithelium; GE, glandular epithelium; S, stroma. Scale bars: 20 μm. Data are expressed as mean ± SEM. **p < 0.01.

The levels of E2-regulated genes were also analyzed. As depicted in Figures 5A–E, the levels of *Esr1*, which encodes the estrogen receptor, and E2-regulated genes, mucin 1 (*Muc1*), lactoferrin (*Ltf*), lipocalin 2 (*Lcn2*), and chloride channel accessory 2 (*Clca3*) were all significantly reduced in cKO uteri. Figures 5F–H illustrates that normal LE, GE, and stroma expressed more ERα, whereas there was minimal ERα expression observed in the epithelium and stroma of cKO uteri. Together, these findings indicate the presence of sustainedly increased P4-responsive genes and decreased E2-responsive genes in cKO uteri.

### *Dcaf2* cKO mice exhibit abnormal genes involved in cell cycle checkpoint, DNA replication, DNA damage, and cell apoptosis

**FIGURE 6:**
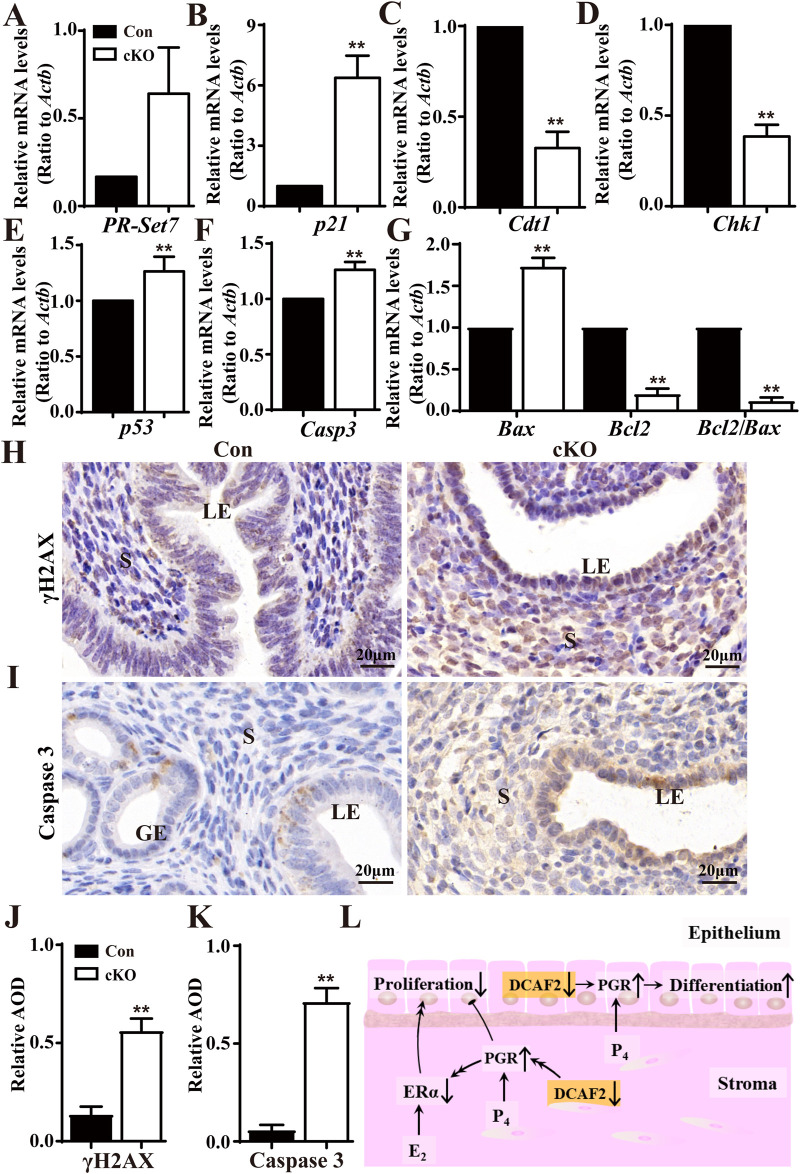
Dysregulated genes involved in cell cycle regulation, histone modification, DNA replication, and cell apoptosis pathway. **(A–D)** QPCR analysis of the substrate levels of CRL4^Cdt2^ complex *PR-Set7*, *P21*, *CDT1*, and *CHK1* in the uteri of Con and cKO mice (n = 6), respectively. **(E–G)** QPCR analysis of cell cycle-associated gene *p53,* and cell apoptosis-associated genes *Casp3*, *Bax,* and *Bcl2* in the uteri of Con and cKO mice (n = 6). **(H,I)** Immunodetecion of DNA damage marker γH2AX and Caspase 3 in the uteri of Con and cKO mice (n = 4), respectively. **(J,K)** Quantification of the immunostaining of γH2AX and Caspase 3 (n = 4), respectively. AOD: Average optical density (IOD [Integrated Optical Density]/area). **(L)** A pattern showing how *Dcaf2* deletion in the uterus affects its development and function. *Dcaf2* deletion leads to increased levels of stromal and epithelial PGR through the CRL4^DCAF2^-mediated ubiquitinated complex, which may reduce the expressions of stromal and epithelial ERα via some undiscovered regulatory mechanisms. Combined with the previous findings (Zhang S. et al., 2013), these dysregulations disrupt cell proliferation and promote the differentiation of epithelial cells, resulting in the absence of glands in the stroma and infertility in cKO mice. LE, luminal epithelium; GE, glandular epithelium; S, stroma. Scale bars: 20 μm. Data are expressed as mean ± SEM. **p < 0.01.

Considering the roles played by DCAF2 depend on substrate degradation, the levels of *PR-Set7*, *P21*, *Cdt1*, and *Chk1* were analyzed. As shown in Figures 6A–D, there were significant increases in the levels of *PR-Set7* and *p21* in cKO uteri compare to Con uteri. Unexpectedly, the levels of *Cdt1* and *Chk1*, two genes encoding well-studied substrates, were notably reduced after *Dcaf2* depletion. Additionally, the levels of *P53* were significantly increased (Figure 6E). Cell apoptosis-associated genes *Casp3* and *Bax* were upregulated, by contrast, *Bcl2* levels were downregulated, resulting in a notable decrease in the ratio of Bcl2 to Bax (Figures 6F, G). It has been reported that *Dcaf2* depletion leads to massive DNA damage, as demonstrated by increased phosphorylated histone H2AX (γH2AX) foci in oocytes (Xu et al., 2017). Here, immunostaining revealed an increase in γH2AX in cKO uteri (Figures 6H, J). The caspase 3 staining depicted in Figures 6I, K was stronger in cKO uteri. These results indicate that deficiency in *Dcaf2* causes abnormal gene expression, specifically in cell cycle checkpoint, DNA replication, DNA damage, and cell apoptosis pathways. As a result, the uterine-specific ablation of *Dcaf2* disrupted the expression of PGR and ERα, consequently, the proliferation and differentiation of epithelial cells were disturbed, and the uterine glands were absent (Figure 6L).

## Discussion

In this study, we aimed to explore the uterine function of DCAF2 by using a mouse model in which DCAF2 was conditionally depleted in the uterus. The breeding study revealed that cKO mice were completely infertile, as their uteri were unable to support embryo implantation. No abnormalities in ovarian structure and function were found in cKO mice. Surprisingly, the cKO mice had severely thin uterus, abnormal epithelium development, and a lack of glands. The transcriptomic data and confirmatory experiments have deciphered how *Dcaf*2 depletion led to these abnormal changes in the uterine epithelial layers. Specifically, it led to the upregulation of PGR and its responsive genes, as well as the downregulation of ERα and its responsive genes. These changes likely explain the infertility observed in the study. Overall, these results suggest DCAF2 plays a crucial role in the development and function of uterine tissue.

DCAF2 plays necessary roles during multiple developmental events, including oogenesis and embryogenesis (Liu et al., 2007; Xu et al., 2017; Yang et al., 2022). *Dcaf2*-depleted oocytes were arrested at the one-to two-cell stage, resulting in infertility (Xu et al., 2017). Depletion of *Dcaf2* in placental trophoblasts caused embryonic death at the gastrulation stage (Yang et al., 2022). In the present study, to elucidate the uterine function of DCAF2, *Pgr* ^Cre/+^ mouse model was used to deplete *Dcaf2* in the uterus. The female cKO mice were sterile. The typical morphological features of these cKO mice include a severely thin uterus, without branching in mature uterine LE architecture, and the absence of uterine glands. Additionally, the stroma in cKO endometria was markedly decreased in thickness and showed hypotrophy. It is well-established that *Pgr* is expressed in all compartments of the uterus, including epithelium and stroma (Soyal et al., 2005). In addition, almost all the expression of DCAF2 was located in the LE and GE. Based on our findings, we concluded that the complete incapability of the uterus to support implantation suggests the loss of fertility in these cKO mice is attributable to defects in the uterine epithelium. Indeed, the reduced or absent uterus glands observed in mice with conditional deletion of epithelia- or glands-derived genes underlie the failure of implantation and thus result in severe subfertility or infertility (Dunlap et al., 2011; Franco et al., 2011). Consistently, our study identifies DCAF2 as another previously unrecognized attractant that is indispensable for the development and function of the glands and the uterus.

As one of the essential events during postnatal uterine development, adenogenesis is guided by intricate mechanisms that regulate morphogenetical cell behaviors including differentiation, proliferation, and movement (Allison Gray et al., 2000). The identification of regulatory factors critical to adenogenesis has greatly benefited from conditional knockout models that disable genes derived from glands. In this study, *Dcaf2* depletion in the uterus inhibited the proliferation of epithelia and caused the loss of glands. Previous research has shown that FOXA2 and WNT7A are critical regulatory factors during adenogenesis (Jeong et al., 2010; Dunlap et al., 2011). Of note, the absence of glands was observed in mice with conditional depletion of *Foxa2* or *Wnt7a* in the uterus. Additionally, in mice lacking ERα or CYP19, an aromatase enzyme critical for E2 synthesis, a hypoplastic uterus with fewer uterine glands develops (Lubahn et al., 1993; Hewitt et al., 2020). This observation clearly highlights the relationship between E2 and DCAF2. Poor development of uterine epithelium directly affects its function. *Foxa2*, a developmentally important gene, is expressed exclusively in the GE of the uterus (Besnard et al., 2004). The morphoregulatory factor WNT7A is present only in the uterine epithelium (Miller and Sassoon, 1998). Conditional knockout of *Foxa2* or *Wnt7a* with *Pgr* ^Cre/+^ mice leads to infertility with no signs of implantation (Jeong et al., 2010; Dunlap et al., 2011). However, the glands can be rescued by infecting the uterus of cKO mice with WNT7A (Dunlap et al., 2011). Furthermore, a reduction in the levels of *Foxa2* mRNA is observed in the *Wnt7a* cKO uterus, primarily due to the absence of glands that abundantly produce FOXA2. In our study, the levels of *Foxa2* and two other GE-specific genes, *Wfdc3* and *Spink3,* were also reduced in the *Dcaf2* cKO uterus. In a word, these intrinsic factors secreted by the epithelium, including DCAF2, play indispensable regulatory roles in the development of glands.

Regarding the regulatory roles played by extrinsic P4 and E2 in the development of uterine epithelium, stromal-epithelial communications play obligatory roles (Li et al., 2011). E2 promotes uterine epithelial cell proliferation by binding to ERα in uterine stromal cells. When *Erα* is knocked out in stromal cells, E2 cannot induce epithelial proliferation (Cooke et al., 1997). NCOA6, a co-factor of ERα, promotes ubiquitination and degradation of ERα. Deletion of *Ncoa6* in stromal cells increases the stability of ERα, leading to excessive estrogen response, sustained epithelial cell proliferation, and implantation failure (Kawagoe et al., 2012). The nuclear inducer of the Notch signaling pathway, RBPJ, limits ERα activity. Loss of RBPJ enhances ERα activity, resulting in excessive response to E2 and inducing abnormal implantation and mid-to-late pregnancy miscarriage (Zhang et al., 2014). These studies indicate that expression of ERα in stromal cells is necessary for epithelial cell proliferation. Whereas P4 counteracts E2-induced epithelial cell proliferation by binding to PGR in stromal cells, leading to differentiation of the epithelial cells (Das and Martin, 1973). When *Pgr* is knocked out in stromal cells, P_4_ cannot inhibit the proliferative effect of E2 on epithelium (Kurita et al., 1998). Deletion of the PGR co-chaperone molecule FKBP52 reduces PGR activity, weakening the inhibitory effect of P4 on E2-induced epithelial proliferation and inducing abnormal proliferation of the uterine epithelium during the peri-implantation period, resulting in progesterone resistance (Tranguch et al., 2007). Downstream molecules of PGR, COUP-TFII, and HAND2 participate in mediating PGR pathway in stromal cells and modulate epithelial function (Kurihara et al., 2007; Li et al., 2011). BMI-1 regulates PGR ubiquitination modification, determines PGR activity, and counteracts the proliferative effect of E2 on the epithelium (Xin et al., 2017). Therefore, the elevated uterine PGR signaling and reduced estrogen receptor signaling in *Dcaf2* cKO mice inhibit epithelial proliferation, resulting in severely thin uteri.

Additionally, the communications are mechanistically regulated by many other transcription factors. E2 may promote epithelial cell proliferation by inducing stromal cell expression of paracrine factors such as IGF-1. Blocking stromal cell IGF-1 expression can inhibit the proliferative effects of E2 on the epithelium (Zhu and Pollard, 2007). Increased stromal PGR upregulates morphoregulatory genes, including *Ihh* (Lee et al., 2006) and *COUP-TFII* (Kurihara et al., 2007). On the other hand, decreased epithelial ER downregulates E2-responsive genes, including *Muc1* and *Ltf* (Surveyor et al., 1995). Synergistically, these processes restrict the proliferation of epithelial cells, thereby blocking the adenogenesis. Our results support these findings. In addition, KLF4 and KLF15 have been extensively studied as downstream mediators of P4 and E2 during the proliferation of uterine epithelium (Ray and Pollard, 2012). In response to E2, KLF4 binds to *Mcm2* promoter, leading to G1/S progression and mitosis. While KLF15 transcriptionally downregulates *Mcm7*, thereby blocking cell cycle progression and inhibiting cell proliferation after P4 and E2 treatment. In our study, we observed significant decreases in *Klf4* and *Mcm2* mRNA levels, as well as significant increases in *Klf15* and *Mcm7* mRNA levels in *Dcaf2* cKO mice. These changes in gene expression corresponded with the inhibition of epithelial cell proliferation in *Dcaf2* cKO mice. Normally, PGR and ER expressions are dynamically regulated to keep their balances (Thomas and Gustafsson, 2015). As for why stromal PGR increased in the cKO uterus, we surmised PGR might be a substrate of CRL4^DCAF2^-mediated ubiquitinated complex. Specifically, after *Dcaf2* depletion, this complex was disassembled and lost the abilities of recognition and degradation of PGR, thus leading to an increase in PGR expression. However, further experiments are needed to confirm this hypothesis.

In conclusion, we have demonstrated the key role of DCAF2 in the development and function of the uterus by utilizing mice with conditional ablated *Dcaf2* specifically in the uterus. The uterine-specific ablation of *Dcaf2* results in a defect in epithelium. Additionally, these mice lack uterine glands and exhibit implantation defects, ultimately leading to infertility. Moreover, the deletion of *Dcaf2* disrupts the expression of PGR and ER, highlighting the critical role of *Dcaf2* in uterine development. These findings provide valuable insights into our understanding of the significance of DCAF2 in female reproduction.

## Data Availability

The original contributions presented in the study are included in the article/Supplementary Material, further inquiries can be directed to the corresponding author.

## Supplementary material

SUPPLEMENTARY FIGURE S1 CKO uteri exhibited abnormalities during puberty. **(A–F)** Uteri at different ages, ranging from postnatal day (PND) 5–42, were dissected from Con and cKO mice.

SUPPLEMENTARY FIGURE S2 RNA-seq analysis revealed differentially expressed genes. **(A)** The heatmap illustrates the differences in gene expression between Con and cKO mice. **(B)** Gene Ontology analysis identified the molecular functions of these differentially expressed genes.

## References

[B1] Abbas T. Dutta A. (2011). CRL4Cdt2: master coordinator of cell cycle progression and genome stability. Cell Cycle 10, 241–249. 10.4161/cc.10.2.14530 21212733 PMC3025761

[B2] Allison Gray C. Bartol F. F. Taylor K. M. Wiley A. A. Ramsey W. S. Ott T. L. (2000). Ovine uterine gland knock-out model: effects of gland ablation on the estrous cycle. Biol. Reprod. 62, 448–456. 10.1095/biolreprod62.2.448 10642586

[B3] Besnard V. Wert S. E. Hull W. M. Whitsett J. A. (2004). Immunohistochemical localization of Foxa1 and Foxa2 in mouse embryos and adult tissues. Gene Expr. Patterns 5, 193–208. 10.1016/j.modgep.2004.08.006 15567715

[B4] Chang X. L. Liu L. Wang N. Chen Z. J. Zhang C. (2017). The function of high-density lipoprotein and low-density lipoprotein in the maintenance of mouse ovarian steroid balance. Biol. Reprod. 97, 862–872. 10.1093/biolre/iox134 29092018

[B5] Cooke P. S. Buchanan D. L. Young P. Setiawan T. Brody J. Korach K. S. (1997). Stromal estrogen receptors mediate mitogenic effects of estradiol on uterine epithelium. P Natl. Acad. Sci. U. S. A. 94, 6535–6540. 10.1073/pnas.94.12.6535 PMC21085 9177253

[B6] Cui T. He B. Kong S. Zhou C. Zhang H. Ni Z. (2017). PR-Set7 deficiency limits uterine epithelial population growth hampering postnatal gland formation in mice. Cell Death Differ. 24, 2013–2021. 10.1038/cdd.2017.120 28731465 PMC5686342

[B7] Das R. M. Martin L. (1973). Progesterone inhibition of mouse uterine epithelial proliferation. J. Endocrinol. 59, 205–206. 10.1677/joe.0.0590205 4748527

[B8] Dunlap K. A. Filant J. Hayashi K. Rucker E. B. Song G. Deng J. M. (2011). Postnatal deletion of Wnt7a inhibits uterine gland morphogenesis and compromises adult fertility in mice. Biol. Reprod. 85, 386–396. 10.1095/biolreprod.111.091769 21508348 PMC3142262

[B9] Franco H. L. Dai D. Lee K. Y. Rubel C. A. Roop D. Boerboom D. (2011). WNT4 is a key regulator of normal postnatal uterine development and progesterone signaling during embryo implantation and decidualization in the mouse. Faseb J. 25, 1176–1187. 10.1096/fj.10-175349 21163860 PMC3058697

[B10] Frantsiyants E. M. Bandovkina V. A. Kaplieva I. V. Cheryarina N. D. Trepitaki L. K. Neskubina I. V. (2020). Influence of malignant growth and chronic neurogenic pain on neurosteroid levels in rat brain. Biomeditsinskaya Khimiya. 66, 151–155. 10.18097/PBMC20206602151 32420896

[B11] Hewitt S. C. Marleny C. Grace F. K. Donoghue L. J. Lierz S. L. Wipawee W. (2020). Peri- and postpubertal estrogen exposures of female mice optimize uterine responses later in life. Endocrinology 8. 10.1210/endocr/bqaa081 PMC7417879 32623449

[B12] Higa L. A. Banks D. Wu M. Kobayashi R. Sun H. Zhang H. (2006). L2DTL/CDT2 interacts with the CUL4/DDB1 complex and PCNA and regulates CDT1 proteolysis in response to DNA damage. Cell Cycle 5, 1675–1680. 10.4161/cc.5.15.3149 16861906

[B13] Jeong J. W. Kwak I. Lee K. Y. Kim T. H. Large M. J. Stewart C. L. (2010). Foxa2 is essential for mouse endometrial gland development and fertility. Biol. Reprod. 83, 396–403. 10.1095/biolreprod.109.083154 20484741 PMC2924802

[B14] Kawagoe J. Li Q. Mussi P. Liao L. Lydon J. P. DeMayo F. J. (2012). Nuclear receptor coactivator-6 attenuates uterine estrogen sensitivity to permit embryo implantation. Dev. Cell 23, 858–865. 10.1016/j.devcel.2012.09.002 23079602 PMC3479668

[B15] Kim Y. S. Yuan J. Dewar A. Borg J. P. Threadgill D. W. Sun X. (2023). An unanticipated discourse of HB-EGF with VANGL2 signaling during embryo implantation. Proc. Natl. Acad. Sci. U. S. A. 120, e2302937120. 10.1073/pnas.2302937120 37155852 PMC10193979

[B16] Knoedler J. R. Denver R. J. (2014). Kruppel-like factors are effectors of nuclear receptor signaling. Gen. Comp. Endocrinol. 203, 49–59. 10.1016/j.ygcen.2014.03.003 24642391 PMC4339045

[B17] Kobayashi A. Behringer R. R. (2003). Developmental genetics of the female reproductive tract in mammals. Nat. Rev. Genet. 4, 969–980. 10.1038/nrg1225 14631357

[B18] Kurihara I. Lee D. K. Petit F. G. Jeong J. Lee K. Lydon J. P. (2007). COUP-TFII mediates progesterone regulation of uterine implantation by controlling ER activity. Plos Genet. 3, e102–e1064. 10.1371/journal.pgen.0030102 17590085 PMC1892047

[B19] Kurita T. Young P. Brody J. R. Lydon J. P. O'Malley B. W. Cunha G. R. (1998). Stromal progesterone receptors mediate the inhibitory effects of progesterone on estrogen-induced uterine epithelial cell deoxyribonucleic acid synthesis. Endocrinology 139, 4708–4713. 10.1210/endo.139.11.6317 9794483

[B20] Lee K. Jeong J. Kwak I. Yu C. T. Lanske B. Soegiarto D. W. (2006). Indian hedgehog is a major mediator of progesterone signaling in the mouse uterus. Nat. Genet. 38, 1204–1209. 10.1038/ng1874 16951680

[B21] Li Q. Kannan A. DeMayo F. J. Lydon J. P. Cooke P. S. Yamagishi H. (2011). The antiproliferative action of progesterone in uterine epithelium is mediated by Hand2. Science 331, 912–916. 10.1126/science.1197454 21330545 PMC3320855

[B22] Liu C.-L. Yu I.-S. Pan H.-W. Lin S.-W. Hsu H.-C. (2007). L2dtl is essential for cell survival and nuclear division in early mouse embryonic development. J. Biol. Chem. 282, 1109–1118. 10.1074/jbc.M606535200 17107960

[B23] Lubahn D. B. Moyer J. S. Golding T. S. Couse J. F. Smithies K. O. Smithies O. (1993). Alteration of reproductive function but not prenatal sexual development after insertional disruption of the mouse estrogen receptor gene. Proc. Natl. Acad. Sci. U. S. A. 90, 11162–11166. 10.1073/pnas.90.23.11162 8248223 PMC47942

[B24] Miller C. Sassoon D. A. (1998). Wnt-7a maintains appropriate uterine patterning during the development of the mouse female reproductive tract. Development 125, 3201–3211. 10.1242/dev.125.16.3201 9671592

[B25] Ray S. Pollard J. W. (2012). KLF15 negatively regulates estrogen-induced epithelial cell proliferation by inhibition of DNA replication licensing. Proc. Natl. Acad. Sci. U. S. A. 109, E1334–E1343. 10.1073/pnas.1118515109 22538816 PMC3361390

[B26] Soyal S. M. Mukherjee A. Lee K. Y. Li J. Li H. DeMayo F. J. (2005). Cre-mediated recombination in cell lineages that express the progesterone receptor. Genesis 41, 58–66. 10.1002/gene.20098 15682389

[B27] Spencer T. E. (2014). Biological roles of uterine glands in pregnancy. Semin. Reprod. Med. 32, 346–357. 10.1055/s-0034-1376354 24959816 PMC4198167

[B28] Surveyor G. A. Gendler S. J. Pemberton L. Das S. K. Chakraborty I. Julian J. (1995). Expression and steroid hormonal control of Muc-1 in the mouse uterus. Endocrinology 136, 3639–3647. 10.1210/endo.136.8.7628404 7628404

[B29] Thomas C. Gustafsson J. A. (2015). Progesterone receptor-estrogen receptor crosstalk: a novel insight. Trends Endocrinol. Metab. 26, 453–454. 10.1016/j.tem.2015.08.002 26277479

[B30] Tranguch S. Wang H. Daikoku T. Xie H. Smith D. F. Dey S. K. (2007). FKBP52 deficiency–conferred uterine progesterone resistance is genetic background and pregnancy stage specific. J. Clin. Invest 117, 1824–1834. 10.1172/jci31622 17571166 PMC1888571

[B31] Wang H. B. Dey S. K. (2006). Roadmap to embryo implantation: clues from mouse models. Nat. Rev. Genet. 7, 185–199. 10.1038/nrg1808 16485018

[B32] Wang N. Q. Li H. Zhu Y. Q. Li N. Chen Z. J. Zhang C. (2020). Melatonin protects against Epirubicin-induced ovarian damage. J. Reprod. Dev. 66, 19–27. 10.1262/jrd.2019-085 31735743 PMC7040211

[B33] Xin Q. Kong S. Yan J. Qiu J. He B. Zhou C. (2017). Polycomb subunit BMI1 determines uterine progesterone responsiveness essential for normal embryo implantation. J. Clin. Invest 128, 175–189. 10.1172/jci92862 29202468 PMC5749512

[B34] Xu Y.-W. Cao L.-R. Wang M. Xu Y. Wu X. Liu J. (2017). Maternal DCAF2 is crucial for maintenance of genome stability during the first cell cycle in mice. J. Cell Sci. 130, 3297–3307. 10.1242/jcs.206664 28818995

[B35] Yang M. Liu M. Wang Z. Zhang C. (2022). Mice lacking DCAF2 in placenta die at the gastrulation stage. Cell Tissue Res. 389, 559–572. 10.1007/s00441-022-03655-4 35711069

[B36] Zhang C. Large M. J. Duggavathi R. DeMayo F. J. Lydon J. P. Schoonjans K. (2013a). Liver receptor homolog-1 is essential for pregnancy. Nat. Med. 19, 1061–1066. 10.1038/nm.3192 23817023 PMC3983050

[B37] Zhang D. Chang X. Bai J. Chen Z. J. Li W. P. Zhang C. (2016). The study of cyclooxygenase 2 in human decidua of preeclampsia. Biol. Reprod. 95, 56. 10.1095/biolreprod.115.138263 27465134

[B38] Zhang S. Kong S. Wang B. Cheng X. Chen Y. Wu W. (2014). Uterine Rbpj is required for embryonic-uterine orientation and decidual remodeling via Notch pathway-independent and -dependent mechanisms. Cell Res. 24, 925–942. 10.1038/cr.2014.82 24971735 PMC4123295

[B39] Zhang S. Lin H. Kong S. Wang S. Wang H. Wang H. (2013b). Physiological and molecular determinants of embryo implantation. Mol. Asp. Med. 34, 939–980. 10.1016/j.mam.2012.12.011 PMC4278353 23290997

[B40] Zhang Y. Yang J. Shijian Z. Zhao D. Q. Chen Z. J. Li W. P. (2018). Downregulation of decidual SP1 and P300 is associated with severe preeclampsia. J. Mol. Endocrinol. 60, 133–143. 10.1530/JME-17-0180 29273682

[B41] Zhu L. Pollard J. W. (2007). Estradiol-17beta regulates mouse uterine epithelial cell proliferation through insulin-like growth factor 1 signaling. Proc. Natl. Acad. Sci. U. S. A. 104, 15847–15851. 10.1073/pnas.0705749104 17895382 PMC2000402

