# Supplementary material for: DCAF2 is essential for the development of uterine epithelia and mouse fertility
Source: Front Cell Dev Biol. 2024 Sep 19;12:1474660. doi: 10.3389/fcell.2024.1474660 (PMC11446810; doi:10.3389/fcell.2024.1474660)
Supplement: Supplementary file 1 [file Table1.docx]

**Supplementary Table 1.** Primer sequences.

| **Gene** | **Sequences (5′-3′)** | **Sequences (3′-5′)** |
| --- | --- | --- |
| *Dcaf2* | TTCTCCTGTCAGTCCTTATG | CAACAACCAGTTCTTATTCTCT |
| *Krt18* | AATATGAAGCCCTCTTGAACA | GCATCGTTGAGACTGAAATC |
| *Cdh1* | AAGACTAGGCTTATCTCAACCAAT | TACCATCAAGAGCAGGCATT |
| *Foxa2* | CGGACCAGGAGAAAGGAGAA | AGCCATAAATAAAGCACGCAGAA |
| *Spink3* | GACGGAATTACTTATGCCAATGA | ATTACGGTAGCCATAACAGAGT |
| *Wfdc3* | TTCCTCGTATCTACTGCTAC | CTACACAGTACAGACAAGAG |
| *Ki-67* | AAGAAGTTAAGAACAAGAAG | ACACTGGAACTTATCACTAA |
| *Klf4* | AAGGAAGAGTTCAAGAGACA | AAGATCCATCCAGTATCAGA |
| *Mcm2* | TTCAGGTGACAGACTTTATC | CTTTGAGAATGTTCTGGAAA |
| *Klf15* | TCTCTCTCAAGGTTTTAG | GGATTGCTTATCATTACC |
| *Mcm7* | AGGAAATGAAGATTCAAGAG | AATTCTTGTGTTCTCACCTT |
| *Pgr* | AGTGAGTTCTTTAGCACATTT | AAGCCATTATGATTTCCACAT |
| *Ihh* | TCACCCTATTTATTTGC | CAAAAGAAAGACCAAAAC |
| *Nr2f2* | TTTAAGGAGATTGGGAGA | CATAGACACAGGACAATT |
| *Hand2* | TGCATATGTGAAGTGTAT | CTGTTTTCTGCTTTGAAA |
| *Areg* | TTCTTTCAGAAAAGGAAG | ACAGAAGTAAGACTATCA |
| *Esr1* | AATAAGCGACTTACTGAT | CAATGACCTTTCAATGTA |
| *Ltf* | CTCAAAGTTTATACTGGA | AACTTCAGGATAAAGGAA |
| *Muc1* | TTACTATAGCACAGTACCAT | AAAGACAAGAAGAAGAAGGA |
| *Lcn2* | AACAATAGCTACAATGTC | AAATGTTCTGATCCAGTA |
| *Clca3* | ATTGTGTATTTGTACCAG | CCACAGAATTGATATTTTG |
| *p21* | AGTCTCCAAACTTAAAGT | GTCCTACTAATTTAGGTT |
| *Cdt1* | TGGAGAAGGCCCTGAGCAACC | CTCTGGCAGACGCTCTAACCGC |
| *Chk1* | AATTTGGTAGAAATGGATG | TGTCTCTTGAACTCTAAT |
| *PR-Set7* | GCCTTATATTACCAAAACTT | TCAACTCCATTCTCTAAAAC |
| *P53* | TTTCTGTCTTCCTATAGCTA | CTTTCTTGACCATTGTTT |
| *Bax* | ACTGTGTCTTTTCTTCATAA | CCCTCCCAATAATTACAAAA |
| *Bcl2* | TGTAGTTTGGTTCTATTTGA | ATAGATAATCTCCACATTCC |
| *Casp3* | AAGTAACTGTCAATGATG | ATTCATAGTATCGCCAAA |
