# Supplementary figures and images for: DCAF2 is essential for the development of uterine epithelia and mouse fertility
Source: Front Cell Dev Biol. 2024 Sep 19;12:1474660. doi: 10.3389/fcell.2024.1474660 (PMC11446810; doi:10.3389/fcell.2024.1474660)

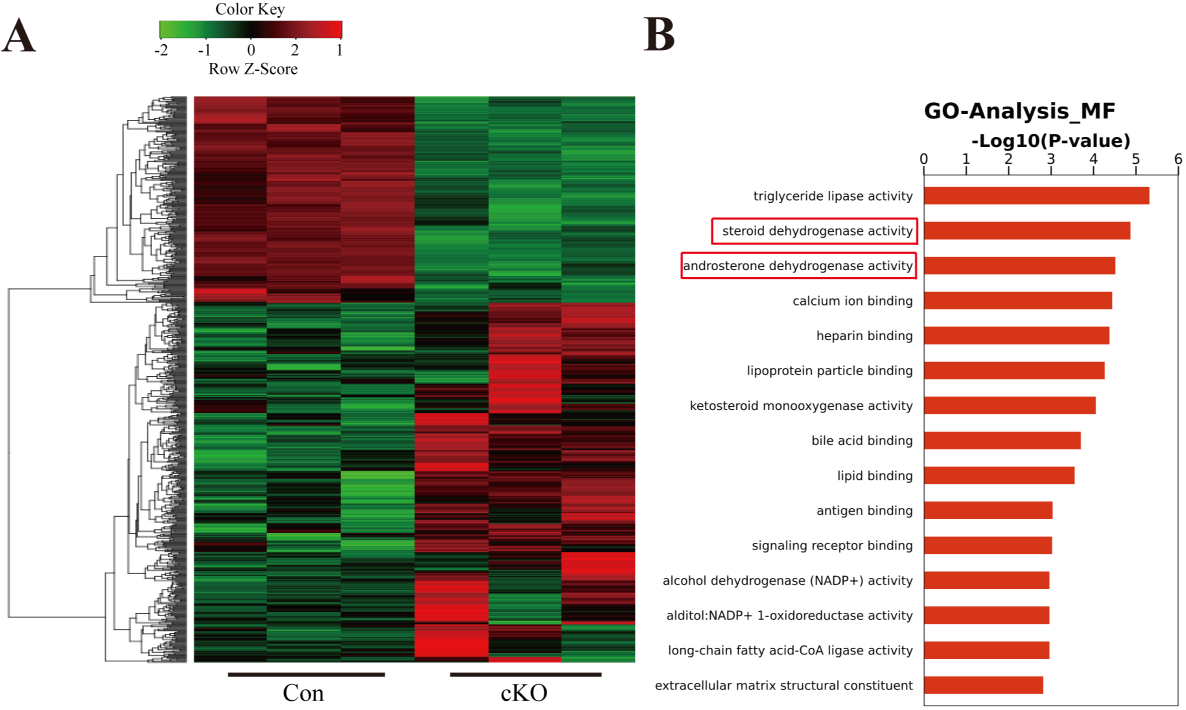

Supplement: Supplementary file 2 [file Image2.TIF]

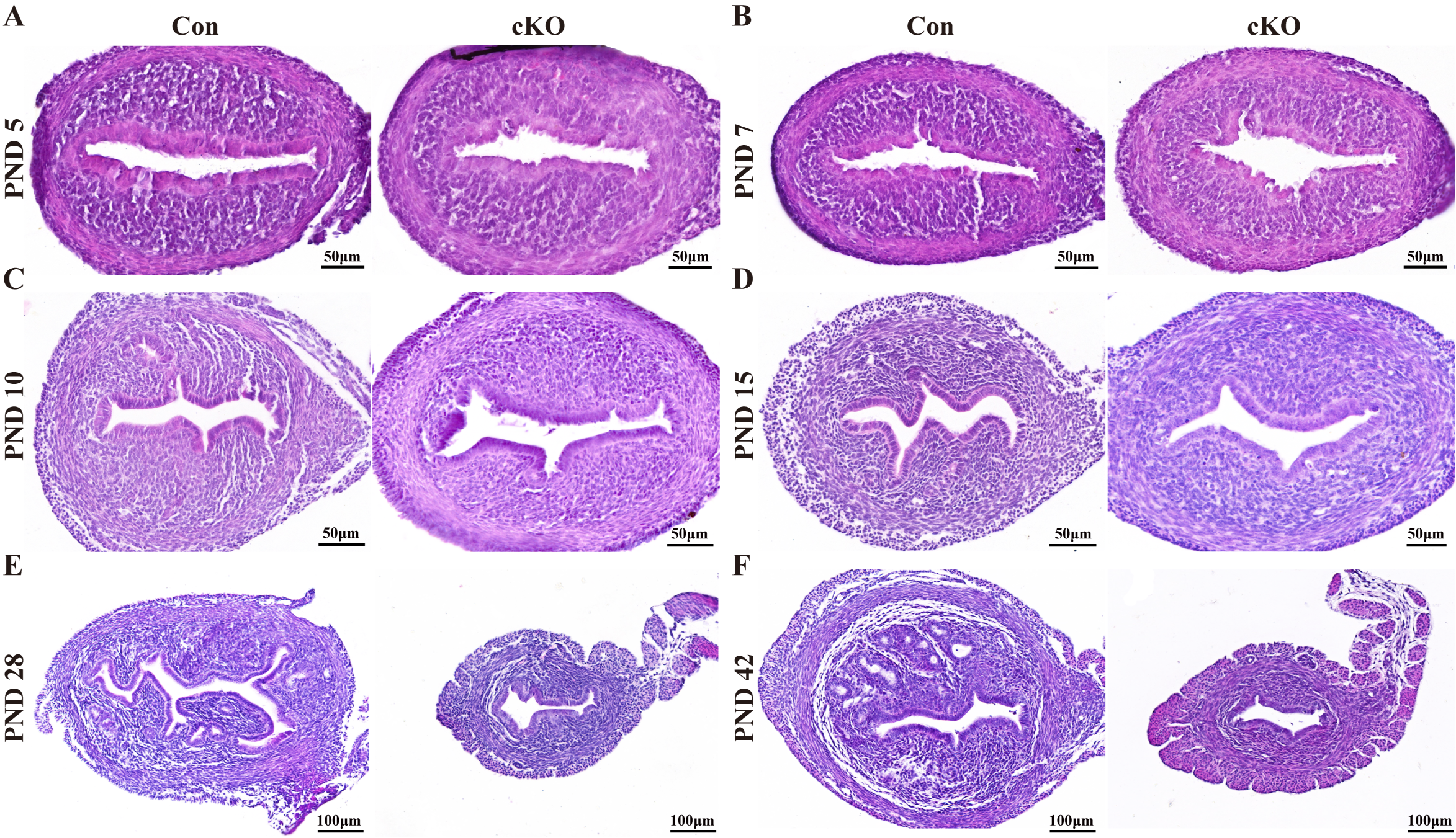

Supplement: Supplementary file 3 [file Image1.TIF]
