# Supplementary material for: DCAF2 is essential for the development of uterine epithelia and mouse fertility
Source: Front Cell Dev Biol. 2024 Sep 19;12:1474660. doi: 10.3389/fcell.2024.1474660 (PMC11446810; doi:10.3389/fcell.2024.1474660)
Supplement: Supplementary file 4 [file Table2.docx]

**Supplementary Table 2.** Antibody information.

| **Protein** | **Manufacturer** | **Catalogue number** | **Working dilution** |
| --- | --- | --- | --- |
| DCAF2 | Abcam | ab72264 | 1:100 |
| SMA | Proteintech | 67735-1-Ig | 1:200 |
| CK7 | Abcam | ab181598 | 1:400 |
| E-cadherin | Abcam | ab231303 | 1:200 |
| Ki-67 | Proteintech | 27309-1-AP | 1:200 |
| PGR | Abcam | ab101688 | 1:400 |
| ERα | Proteintech | 21244-1-AP | 1:200 |
| γH2AX | Huabio | ER1901-70 | 1:200 |
| Caspase 3 | Huabio | ER30804 | 1:200 |
